# Predicting student mental health through entropy-based features and interpretable cross-attention transformer networks

**DOI:** 10.1371/journal.pone.0347294

**Published:** 2026-04-21

**Authors:** Dan Jiang

**Affiliations:** School of Language and Culture, Qingdao Huanghai University, Qingdao, Shandong, China; University of Sargodha, PAKISTAN

## Abstract

Mental health is becoming a major concern for students in today’s fast-changing world. Mental health challenges have impact on every aspect of life including performance which pointed to early identification of risk levels. Recent reports show a rapid rise in anxiety, depression, and stress among students. This need urge for intelligent systems that can support mental health monitoring in educational environments. Existing methods often lack accuracy, and the ability to capture complex psychological patterns. This study aims to address these gaps by developing an interpretable deep learning model that predicts student mental health risk using FT-Transformer and LSTM architectures. The model integrates a Cross-Attention Attribution Layer (CAAL), which combines feature attention with temporal attention, making the ensemble intrinsically interpretable. The approach captures both global feature relationships and time-based emotional variations. Feature engineering based on entropy and uncertainty patterns further strengthens the model’s ability to detect subtle risk signals. The proposed method is compared with several baseline models, including SVM, Logistic Regression, Random Forest, standalone LSTM, and FT-Transformer. Empirical analysis shows that the proposed model achieves the highest accuracy of 95%, outperforming all baselines. These findings are validated through explainable AI techniques, global feature-importance analysis, and multiple statistical tests for effective framework to support student mental health assessment.

## Introduction

Psychological well-being plays a key role in students’ academic and personal success. With increasing stressors from academics, lifestyle changes, and social environments, students often face emotional struggles that go unnoticed and then cause of stress [1]. These pressures influence emotional balance and academic performance. Growing evidence shows that mental health problems are increasing among young learners and need timely attention [2]. Early identification of mental health risks is essential to support timely help. Traditional tools often miss subtle signals, making room for smarter, data-driven systems.

**Fig 1:**
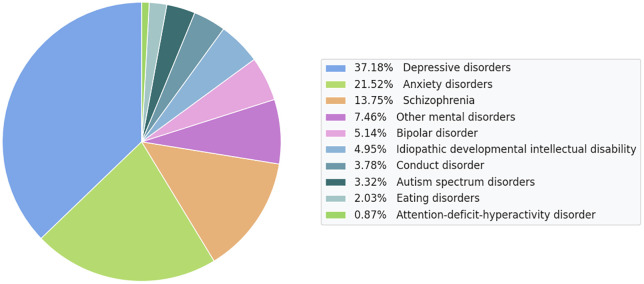
Distribution of global mental health disorder prevalence illustrates the relative proportions of major mental health conditions [5].

Changes in lifestyle and learning patterns make students more vulnerable to stress. Many face anxiety, depression and low motivation [3]. These issues reduce participation in classes and affect long term progress. Poor mental health harms self-esteem and limits the ability to manage daily tasks. It also affects relationships and overall, well-being [4]. The global analysis as shown in Fig 1, highlights that depressive disorders (37.18%) and anxiety disorders (21.52%) are the most prevalent mental health conditions. Other significant issues include schizophrenia, bipolar disorder, and developmental disabilities, indicating a wide spectrum of psychological challenges affecting populations worldwide [5].

The recent advances in the field of artificial intelligence offer an opportunity to detect the patterns of mental health [6]. The complex psychological cues that are hard to observe using the conventional approaches can be picked by machine learning models. High-level architecture can acquire experience of interaction between features and give early indications of risk [7]. Such systems assist teachers and counselors and establish a background of preventive intervention [8].

The traditional models of machine learning and simple deep learning networks are frequently used in traditional models of mental health prediction. Most of these techniques are very reliant on manual feature engineering and statistical correlations [9]. They have trouble in detecting subtle trends in mental health information since mental health cues are complicated and typically nonlinear. Other models like Logistic Regression and SVM do not take into consideration the interaction between features [10]. Basic deep learning models such as CNNs and simple LSTMs are only able to capture a small amount of either time or structure. Conventional methods often lack interpretability, limiting trust in high-stakes applications especially in nursing-healthcare [11]. The majority of studies present are also not interpretable, and this is essential in mental health research since predictions should be clear and credible [12]. Additionally, many models do not capture temporal and feature-wise dependencies effectively, overlooking complex behavioral patterns. Most of these models give only the values of accuracy but the reason as to why a student is predicted to be at high risk is not given. This leaves a significant divide in practice [13]. Hence, traditional mental health assessment methods rely heavily on subjective reporting, which may lead to underdiagnosis or delayed intervention. This study addresses these gaps through an interpretable and robust deep learning-based framework.

The current analysis addresses these gaps by providing a hybrid interpretable model that learns patterns of features and changes over time jointly and explaining them through the application of cross attention and explainable AI techniques.

The aim of this study is to build an interpretable model for student mental health risk prediction. The model uses FT Transformer and LSTM to learn feature interactions and temporal behavior. It also includes a Cross Attention Attribution Layer that explains how each part of the input contributes to predictions. The main contributions are:

- This study proposes an interpretable FT-Transformer + LSTM hybrid model that achieves 95% accuracy for student mental health risk prediction.
- The study presents a complete feature engineering framework based on entropy, which improves the detection of subtle psychological variations.
- The model is supported with detailed explainable AI analysis using LIME, providing instance-level reasoning for each prediction.
- The study includes statistical validation to confirm model reliability, ensuring that performance improvements are significant and stable across different tests.

This paper is organized as follows. Related work section reviews existing studies on mental health prediction. The proposed method is then presented. The data analysis is provided and then results follows. The paper ends with the conclusion and future work.

## Related work

Social media has emerged as a formidable tool of tracking psychological health in recent years, particularly in college students. In recent years, scientists have been applying machine and deep learning methods to study the content created by users and determine the indicators of mental health problems, including depression, anxiety, and stress [14]. This section will also examine the review of the model, methodologies, datasets, and findings applied to examine mental health using social media.

The study explored a preliminary discussion of AI in mental health and mentioned that data-driven AI approaches can extract behavioral or mental health insights using the digital exhaust of an individual [15]. Furthermore, the advantages and disadvantages of social media in mental health research and propose online tools as the means to reach the maximum opportunities to identify and respond to psychological distress [16]. In a critical review of predictive methods of mental health status, highlighted the advancements in NLP-based mood prediction, but warn that there exists significant bias and generalizability problems [17]. The study analyzed the studies that use ML to detect depression on social media text. They concluded that machine learning methods can relatively predict the outcomes of depression but focus on the data source variation and the necessity of conventional assessment [18]. A scoping review of the ML models to detect anxiety and depression on social media. They have noted that most works deal with depression recognition via Twitter, and they tend to utilize English data, and they identified such methodological issues as an unbalanced dataset and a lack of consideration of linguistic nuances [19]. Likewise, another a systematic audit of bias of the social-media-based mental illness classifiers. They discovered that they depended heavily on Twitter and that the main problems encountered included non-representative samples, irregular model tuning, and diffuse consideration of language nuances [20]. The multimodal framework related to the text, image, and user interaction characteristics aimed at identifying depression behavior based on Twitter data [21]. Likewise, another study concentrated on suicidal thoughts recognition in the Spanish social media. They suggested a combination of textual signals, user relations, and posting patterns to determine risk of suicide and it was proved that a multimodal ML model may be more successful in detecting at-risk users than single-source predictors [22].

There has also been active use of deep learning. A deep RNN based on LSTM to detect depression using textual data. With a Twitter dataset, their two-layer LSTM network did 88% in the classification of users as depressed vs. control. Although this is very high, the outcome indicates that customized deep architectures have the capacity to identify linguistic features of mental illness [23]. The use of lexicon approach with machine learning in early recognition of depression. They constructed language-specific lexicons of depression and a two-step classifier to initially intercept posts which were related to depression on Twitter and subsequently tested it on a university student board [24]. The CNN and LSTM typically used in the study were combined with the purpose of recognizing depressive samples based on multi-source social media text [25]. The Mixture-of-Experts model with BERT embeddings used to predict depression and anxiety based on self-reports to Brazilian social media. They performed better than their traditional feature-based models, which showed the usefulness of professional model ensembles in the identification of various mental health signals [26]. The language models based on transformers to classify depressive posts and they stated that fine-tuned variants of BERT had higher recall of depressive posts than previous architectures relied on RNNs or n-grams [27]. AI-based application that forecasts severe mental distress in college students based on demographic, lifestyle, and habits data. They fitted some ML algorithms on features of the survey; the best of those models (eXGBM) has an AUC of 0.932, an 85.0% accuracy, and an F1-score of 0.856 in classifying high-distress individuals [28]. In another study, the social media content of college students: they developed a new NLP system that incorporated a RoBERTa transformer and GRU recurrent layers and multimodal features [29].

The other course of action considers platforms and behaviors that are popular among the youth. Research investigated Tik Tok and its impact on the mental health of university students and concluded that problematic Tik Tok promotion is associated with a greater level of depression and insomnia in students (p < 0.001) [30]. Statistical modeling to demonstrate the effect of social media addiction on depression and emphasize the type of input features, which the ML-based methods can utilize to predict mental health [31]. On the same note, a quasi-experimental study of the U.S. college networks and demonstrated that the emergence of Facebook resulted in major drops in the mental health of students [32]. The traditional ML models directly to deep learning models in the classification of mental health conditions based on text on social media. They discovered that when it came to medium sized datasets, the two methods had similar accuracies [33]. The gist of high-accuracy models by incorporating explainable AI (XAI) methods. They used LIME to detect depression on 28 different combinations of classifiers and features systematically. This method offered practical information to the mental health practitioners as it harmonized the extremes of rawness and clinical utility [34]. The KC-Net (Knowledge-aware Contrastive Network) designed to detect early stress/depression. In this model, mental health knowledge graphs and contrastive learning were added to improve the detection performance and semantically significant features raised by the model [35]. A multi-modal BiLSTM network that incorporates both the linguistic and the posting timestamps in identifying the early evidence of mental health crisis. In addition to content, modeling the posts of a user over time also resulted in an accuracy of their DABLNet of about 75.9% on Reddit data to classify mental health state [36]. The viewed the time aspect at the population level to an even greater extent which they utilized time-series emotion indicators on Twitter that can predict daily demand of mental health services in Singapore. Such new application of ML connecting social mood to the health of the population implies that social media cues can be used by campus counseling or hospital as an early warning system [37].

Recent studies underscore the rising urgency of mental health concerns and the limitations of traditional diagnostic methods, which have prompted exploration into advanced technologies such as AI and ML. The study highlights the global mental health crisis and advocates for AI-driven solutions to enhance diagnosis and treatment efficacy [38]. Complementing this, another study reviews 184 works on ML applications using multimodal data, emphasizing the importance of non-intrusive data collection and neural network-based feature fusion to capture complex behavioral patterns [39]. Meanwhile, study addresses the critical issue of model interpretability in healthcare, promoting the use of XAI techniques to ensure transparency and trust in clinical applications [40]. Together, these works illustrate the evolution toward interpretable, data-driven mental health frameworks capable of personalized and ethical care.

The recent interdisciplinary studies have also contributed a good deal to the understanding of mental health under various dimensions such as physiological, environmental, behavioral as well as neuropsychological dimensions. E.g. strain-based metrics to predict traumatic brain injury (TBI) [41] highlight the importance of accurate modeling when evaluating at the neural level in mental health. On the same note, the perception of thermal comfort was demonstrated to be moderated by psychological conditions in the case of environmental stressors [42], indicating that emotion-based models are fundamental in creating student-focused wellbeing settings. It has been discovered that information feedback can be used to deactivate cognitive bias and decision inertia in consumer behavior, which provides information on how people respond habitually during moments of emotional stress [43]. A new ensemble method of automatic depression diagnosis is developed in clinical detection to combine emotion-specific modeling with multiscale data processing that, unlike single-scale analysis, can be applied in mental health diagnostics [44]. The pattern of smartphone usage is also an indication of intricate psychological regulation that involves individual belief in control as a mediator of emotional influence on the digital activity [45]. Finally, the inter-brain synchrony studies provide novel information on the neural processes of teamwork and stress, with the neural efficiency being a mental health data point regarding the high-demand team environment [46]. Taken together, these results highlight the importance of multimodal, context-sensitive, and interpretable models to identify the complex interplay of mental health research variables, in terms of cognitive, emotional and behavioral variables.

### Limitations of prior studies

Despite notable advancements, existing studies face several limitations. Many rely on small or platform-specific datasets, which restrict generalizability across diverse student populations. Several models focus heavily on accuracy without incorporating explainability, making them difficult to interpret or deploy in real-world mental health interventions [47]. Additionally, most approaches treat features independently, overlooking the temporal or interactive effects of psychological attributes [48]. To address these gaps, this study introduces an interpretable FT-Transformer + LSTM ensemble that models both feature-level and sequential dependencies, integrates uncertainty-aware feature engineering, and applies explainable AI methods to offer transparent, reliable predictions for student mental health risks. This architecture not only enhances predictive accuracy but also offers transparent decision explanations, ensuring responsible AI usage in educational psychology. Table 1 shows the summary of related work on social media influence and mental health, on college student populations focusing on their approaches and findings.

**Table 1:** Summary of prior studies on mental health.

Refs	Approach	Techniques	Data	Strengths	Limitations	Research Focus
[21]	Multimodal depression detection	Text + image + user features; statistical fusion	Twitter (English) – ~ 5,000 users	Combines heterogeneous data (posts, images, network) for higher accuracy	Data from one platform; complex model hard to deploy	Detecting depressive users via multi-source social data
[22]	Suicide risk assessment model	NLP + image + relational analysis (multimodal)	Twitter, Instagram (Spanish users)	Holistic view (language + behavior); validated on real at-risk users	Focus on Spanish content limits global generality	Multimodal detection of suicidal ideation on social platforms
[24]	Bibliometric study (ML + mental health)	Network and trend analysis of publications	311 papers (2000–2020)	Reveals research trends (e.g., spike in 2017–2020 work)	No model evaluation – bibliometric scope	Research trends in ML for mental health on social media
[29]	RoBERTa + GRU (multimodal)	Transformer text encoder + recurrent time modeling	Twitter, Reddit posts (college students)	State-of-art NLP with temporal context; high accuracy	Requires multi-platform data; black-box model	*Detecting depression in college students’ social media posts*
[23]	Deep Neural Network (DNN)	2-layer LSTM + dense layers (text)	Twitter (global English posts)	Very high performance on training dataset	Possible overfitting; limited transparency	Early depression detection using deep LSTM on textual data
[48]	Two-step lexicon-based classifier	Depression lexicons (EN/KO/JA) + SVM classifier	Twitter (multi-lingual) + “Everytime” student forum	Culturally adaptable (language-specific lexicons); tested on students	Lexicons need updating; may miss slang or context	Detecting depression posts across languages (incl. college network data)
[35]	KC-Net (Knowledge-aware Contrastive)	Mental health knowledge graph + contrastive learning; BERT base	Reddit and Twitter (stress/depression posts)	Uses domain knowledge to improve feature learning; good early-detection performance	Complex training (needs curated knowledge base)	*Early stress & depression detection with knowledge-enhanced deep learning*
[26]	Mixture-of-Experts + BERT	Ensemble of BERT classifiers (Portuguese)	Local social network (Brazil, self-reports)	Outperforms single-model and n-gram baselines; handles multi-condition classification	Language-specific (Portuguese); requires more data for other languages	Predicting depression and anxiety from social media self-report posts
[6]	Framework & challenges review	Taxonomy of ML methods; risk factor analysis	– (Journal of Healthcare Inform. Research)	Proposes multidimensional framework (features, models, outcomes) for depression detection	No new empirical results; highlights research gaps	Classification framework for depression detection on social media (research agenda)
[47]	Hybrid CNN + LSTM model	Word2Vec embeddings; CNN for features + LSTM sequence modeling	Twitter & Facebook combined dataset	Leverages sequence context and pre-trained word vectors; improved accuracy over ML baselines	Moderate performance; limited interpretability	Detecting depression from multi-platform text via hybrid deep learning
[28]	eXtreme GBM (ensemble tree)	Feature-based ML (demographics, lifestyle)	Survey data (2,088 college students, China)	High AUC; validated externally on other universities (AUC 0.918)	Uses survey/self-report data (not passive social media); limited to severe distress prediction	AI tool to predict severe mental distress in college students (non-social-media features)
[30]	Pre-trained LM + SVM (CWINCA feature select)	BERT embeddings; CWINCA feature selection; SVM classifier	6 datasets (Twitter, Reddit – depression & suicidal content)	Tested across multiple datasets (robustness); very high precision on some sets	Near-100% on some data suggests overfitting or easy dataset; black-box feature selection	Detecting depression and suicidal ideation via BERT features + feature selection ensemble
[36]	DABLNet (BiLSTM + Attention)	BiLSTM text encoder + LSTM temporal encoder + cross-modal attention	Reddit posts (timeline of user posts)	Incorporates posting time patterns; addresses context changes over time	Moderate accuracy; requires longitudinal data per user	Early detection of mental health crises using text + temporal social media features
[34]	Multi-model comparison + XAI	SVM, RF, XGB, ANN (28 combos) with LIME explanations	Twitter (English depression dataset)	Comprehensive evaluation of features and classifiers; provides model explainability (LIME)	Slight drop in accuracy compared to deep models; only evaluated on one dataset	Explainable depression detection – comparing traditional ML vs. deep models with interpretability
[10]	ML vs. DL benchmark	LR, RF, LightGBM vs. BERT (ALBERT) and GRU	Reddit (multi-class: depression, anxiety, control)	Head-to-head evaluation under same conditions; provides guidance on model choice	Models perform similarly – limited gain from complex DL on medium data; interpretability trade-off	Empirical comparison of traditional machine learning vs. deep learning for social media mental health classification
[37]	Time-series forecasting (public health)	Granger-causal analysis; ARIMA with Twitter emotion inputs	Twitter daily emotions + hospital visits (Singapore, 549 days)	Novel use of social media mood to predict population mental health demand; real-world data	Aggregate trends only (not individual prediction); specific to pandemic context	Using Twitter-derived emotions to forecast mental health service needs during a crisis
[13]	SWB classification + growth modeling	SVM classifier for Subjective Well-Being levels; Latent Growth Curve modeling	Sina Weibo posts (1,322 users, China) during COVID-19	Combined ML text analysis with longitudinal modeling; identified heterogeneity in well-being trends	SWB labeling model not fully described; observational study (no intervention)	Assessing college users’ subjective well-being over time from social media content (COVID-19 impact)
[32]	Natural experiment analysis	Difference-in-differences (Facebook rollout in colleges)	2004–06 U.S. college student surveys (Facebook vs non-FB cohorts)	Strong causal evidence linking social media availability to mental health decline in students	No ML or individual prediction; older cohort data	Causal impact of social media introduction on college student mental health (depression, anxiety)

## Research design and workflow

**Fig 2:**
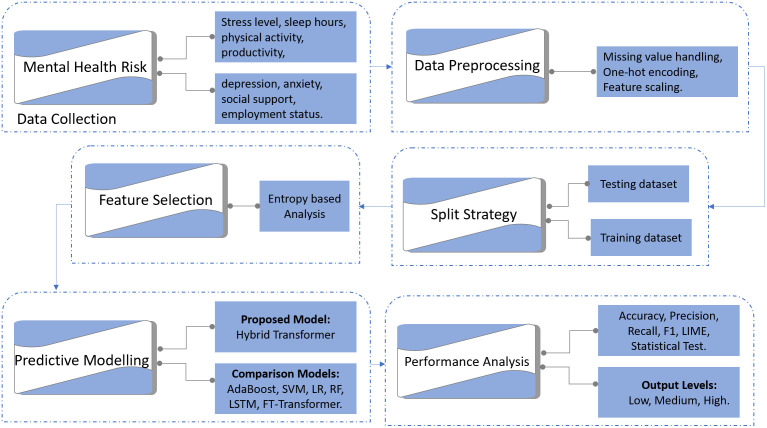
A proposed pipeline illustrating the proposed approach, from data preprocessing to explainable AI analysis for student mental health prediction.

The approach of this study is systematic because it develops a predictive model of student mental health risk that is understandable and accurate. The process is composed of data collection, preprocessing, feature engineering, development of the baseline model, the proposed hybrid model, performance evaluation, interpretability analysis and statistical validation, framework shown in Fig 2. The steps ensures the pipeline be reliable, transparent and useful towards mental health assessment.

### Data acquisition and cleaning process

The data sourced from online repository Kaggle, consisting of 2,000 student records with 10 psychological and behavioral features, such as anxiety score, depression score, productivity level, sleep hours, and physical activity. Each feature was collected via self-reported survey instruments commonly used in psychological assessment studies. The dataset includes both continuous and categorical attributes. For classification, mental health risk was labeled as Low, Medium, and High, based on composite score thresholds derived from normalized anxiety and depression values. Specifically, thresholds were defined using percentile-based segmentation: scores below the 33rd percentile were labeled as Low risk, between 34th–66th percentile as Medium, and above the 67th percentile as High risk. This stratification ensures balanced class representation and reflects increasing psychological distress levels in alignment with established screening guidelines. The target labels *Low, Medium, and High* mental health risk represent varying degrees of psychological distress. *Low* indicates manageable stress levels with minimal disruption to daily life. *Medium* corresponds to moderate symptoms that may impact academic or social performance. *High* signifies severe mental health concerns requiring attention, potentially including anxiety, depression, or burnout indicators. Handling mental health data requires strict ethical considerations. This study ensured anonymization and privacy safeguards to protect individual identities. Moreover, responsible AI practices were followed, promoting transparency and fairness in model predictions. Given the sensitive nature of student wellbeing, such safeguards are vital for building trust and ensuring ethical deployment of predictive systems in academic settings. Preprocessing involved filling in of missing values, correcting erroneous entries and transforming categorical variables into numerical values using encoding. Standard normalization was used to scale all the numerical characteristics since the model was to be trained to be stable. Low, medium or high mental health risk was utilized as the target label. Stratified sampling was then used to divide the dataset into the training and testing sets ensuring classes are balanced.

### Deriving informative feature

The field of feature engineering was concerned with extracting informative patterns of the psychological and behavioral variables. The uncertainty scores based on entropy values were computed using the features of interest to determine variability and inconsistency in emotional conditions [49]. Further modification was made to bring out non-linear relations and enhance the capability of the model to learn risk patterns. The given process aided in determining the most influential predictors and better model sensitivity.

### Comparative baseline approaches

Various baseline models were applied to compare them, such as Logistic Regression, Support Vector Machine, the Random Forest, Naive Bayes, and individual LSTM and the FT-Transformer. The preprocessed dataset was used to train each of the models. These baselines were aimed at comparing the capabilities and drawbacks of traditional and deep learning methods in mental health risk prediction. Their performance was used to evaluate the gains that the proposed model had made.

### Advanced ensemble model design

The model proposes a hybrid system of FT-Transformer and LSTM. FT-Transformer learns the interactions of global features using self-attention and LSTM learns the sequential patterns existing in the scores of psychology. A Cross Attention Attribution Layer (CAAL) directly links both the parts and offers explicit communication between feature-wise attention and temporal attention. This framework assists the model to learn complicated relationships in behavior and be interpretable. The last layer of output makes a prediction of the mental health risk category.

#### Input feature embedding.

This layer transforms every feature of dataset into a single numerical one. Every student is denoted as feature vector with psychological, behavioral and demographic characteristics. This forms the foundation embedding which goes to the attention and sequence layers. Table 2 shows the symbols notations with description.

**Table 2:** Notation and description of symbols used in the proposed model architecture.

Symbol	Meaning
$a_{anx}$ and $a_{dep}$	Anxiety and Depression score
$a_{prod}$ and $a_{soc}$	Productivity and Social support score
$s_{sleep}$ and $s_{stress}$	Sleep and Stress hours
$a_{age}$	Age
$d_{pa}$	Physical activity days
$e_{emp}$ and $g_{gen}$	Employment status and gender encoding
$A_{T}$	transformer feature-attention output
$W_{Q},W_{K},W_{V}$	projection matrices
d	feature dimension
$x_{PE}$	position-enhanced feature vector
i and d	feature index and number of features
$h_{T}$	Final hidden state representing temporal psychological dynamics
$A_{C}$	cross-attention attribution map
$W_{F},W_{L}$	fusion matrices
$h_{Ts}$	LSTM temporal summary
$\hat{y}$	predicted risk category
$W_{O},b_{O}$	classifier parameters


$$x=[a_{anx},\;a_{dep},\;a_{prod},\;a_{soc},s_{sleep},\;a_{age},\;s_{stress},\;d_{pa},\;e_{emp},\;g_{gen}]$$ (1)

#### FT-transformer feature attention layer.

This layer learns which features interact strongly with each other. For example, anxiety and depression often reinforce each other, while productivity and stress have opposite directions. The attention mechanism gives higher weights to psychologically critical features.


$$A_{T}=\text{softmax}\;(\frac{(xW_{Q})(xW_{K}{)}^{⊤}}{\sqrt{d}})(xW_{V})$$ (2)

#### Positional-psychological encoding layer.

This layer imposes a psychological ordering on features. Emotional features are placed first, followed by behavioral features and demographic features. This structured ordering helps the model learn progressive risk patterns.


$$x_{PE}(i)=x(i)+\text{sin }(\frac{i}{{\text{10}}^{\text{2}i/d}})$$ (3)

The ordering of features into Emotional → Behavioral → Demographic categories is informed by both theoretical grounding and empirical considerations. Emotional attributes such as *depression_score* and *anxiety_score* are known to be primary indicators of psychological distress and thus are positioned first to emphasize their immediate influence. Behavioral variables, including *productivity_score*, *sleep_hours*, and *physical_activity_days*, typically manifest as consequences or external expressions of underlying emotional states and are therefore sequenced subsequently. Demographic factors, such as *age*, *gender*, and *employment_status*, while relevant, tend to exert indirect or background influence on mental health and are accordingly placed last. To assess the impact of feature sequencing on model performance, three distinct input orderings were evaluated: (i) Order 1 – *Demographic → Behavioral → Emotional*, (ii) Order 2 – *Behavioral → Emotional → Demographic*, and (iii) Order 3 – *Emotional → Behavioral → Demographic* (Proposed). This structured ordering supports the design of the positional-psychological encoding, enabling the transformer to attend hierarchically based on clinical relevance and temporal sensitivity.

#### LSTM temporal-pattern layer.

The LSTM treats the ordered features as a psychological sequence. It learns how emotional states such as anxiety and depression influence later features like productivity, stress, and social support. This creates a temporal-like emotional-behavior pattern.


$$h_{T}=\text{LSTM}(x_{PE})$$ (4)

#### Cross-Attention Attribution Layer (CAAL).

This layer fuses two types of attention: featured attention from the transformer and interaction-flow attention from the LSTM. It highlights which feature–interaction pairs (e.g., “depression × productivity decline”) contribute most to the final decision. This layer makes the model intrinsically interpretable.


$$A_{C}=\text{softmax}\;(A_{T}W_{F}\cdot \overset{⊤}{\underset{T}{h}}W_{L})$$ (5)

#### Risk classification layer.

The fused representation is passed into a SoftMax classifier. The classifier assigns the student into Low, Medium, or High mental health risk.


$$\hat{y}=\text{softmax}(A_{C}W_{O}+b_{O})$$ (6)

It uses the fused attention features to make a final risk decision.

### Evaluation criteria and scores

All the models were assessed based on accuracy, precision, recall and F1-score. Such measures aided in comparing the predictive quality of models and indicated the gains which the proposed architecture would provide, shown in Table 3. Further, LIME was used to obtain interpretability to model the estimated a particular risk class of a student. It was also provided with intrinsic interpretability by the CAAL layer, which depicted the interaction of features with temporal patterns during prediction. Moreover, The ANOVA, Chi-Square, Wilcoxon, Friedman and Diebold-Mariano were used to statistically validate it [50]. These tests were used to measure significance, consistency and stability of the performance of the model. The findings affirmed the hypothesis that the introduced model did not only outperform baseline approaches but also had reliable improvements on various measurements of statistics.

**Table 3:** Unified Analysis of performance measures, LIME interpretability measures, and statistical validation tests and their mathematical formulas and brief descriptions.

Category	Measure	Formula	Description
Performance Measures	Accuracy	$\text{Acc}=\frac{TP+TN}{TP+TN+FP+FN}$	Overall proportion of correctly classified samples.
	Precision	$\text{Prec}=\frac{TP}{TP+FP}$	How many predicted positive cases are actually positive.
	Recall	$\text{Rec}=\frac{TP}{TP+FN}$	Ability to detect true positive (high-risk) students.
	F1-Score	$F\text{1}=\text{2}\cdot \frac{\text{Prec}\cdot \text{Rec}}{\text{Prec}+\text{Rec}}$	Harmonic mean of precision and recall.
LIME Measures	Local Fidelity (R²)	$R^{\text{2}}=\text{1}-\frac{\sum (y-\hat{y}{)}^{\text{2}}}{\sum (y-\bar{y}{)}^{\text{2}}}$	Shows how well the local surrogate explains the model.
	Positive Contribution Score	$C^{+}=\sum_{i:w_{i}>\text{0}}w_{i}x_{i}$	Total supportive effect of features increasing prediction.
	Negative Contribution Score	$C^{-}=\sum_{i:w_{i}<\text{0}}w_{i}x_{i}$	Total effect of features that lower predicted risk.
	Feature Stability Score	$S=\frac{\text{1}}{N}\sum_{i=\text{1}}^{N}\text{Jaccard}(F_{i},F_{j})$	Consistency of LIME-selected features across instances.
Statistical Tests	ANOVA	$F=\frac{SS_{b}/df_{b}}{SS_{w}/df_{w}}$	Tests difference in predictions across feature groups.
	Chi-Square	$\chi^{\text{2}}=\sum \frac{(O-E{)}^{\text{2}}}{E}$	Measures association between categorical features and risk.
	Wilcoxon Signed-Rank	$W=\sum \text{sgn}(d_{i})\cdot R_{i}$	Compares predicted vs. actual labels using paired ranks.
	Friedman Test	$\overset{\text{2}}{\underset{F}{\chi }}=\frac{\text{1}\text{2}N}{k(k+\text{1})}\sum \overset{\text{2}}{\underset{j}{R}}-\text{3}N(k+\text{1})$	Checks consistency of rankings across model folds.
	Diebold–Mariano Test	$DM=\frac{\bar{d}}{\sqrt{\frac{\text{2}\pi {\hat{f}}_{d}(\text{0})}{T}}}$	Compares forecast error of proposed model vs. baseline.

Although this study utilizes a publicly available dataset sourced from Kaggle, the model’s architecture is designed with adaptability for real-world applications. The proposed FT-Transformer + LSTM ensemble can be integrated with electronic health records (EHR) by mapping structured clinical variables to the model’s input features. In clinical environments, such a model can assist in early mental health risk screening by processing temporal and contextual data from students’ behavioral records or routine assessments. Moreover, the inclusion of explainable AI components (like CAAL and LIME) ensures that model decisions remain transparent to healthcare providers. For real-time deployment, data preprocessing and standardization pipelines can be adapted to suit live data streams while maintaining ethical considerations and data privacy. The proposed algorithm integrates FT-Transformer with LSTM using CAAL to model students’ psychological health from temporal behavioral features. The process begins with feature embeddings passed through LSTM to capture sequential patterns, followed by FT-Transformer to learn feature-level dependencies. CAAL fuses both temporal and feature-level attention, highlighting relevant patterns and interactions. The model is trained using categorical cross-entropy loss, and performance is optimized via hyperparameter tuning. This hybrid structure enables robust classification while supporting interpretability through attention maps.

**Algorithm: Interpretable FT-Transformer + LSTM for Mental health Risk Prediction**

ProposedHybridModel(D)

Input:

Dataset $D=\{(x_{i},y_{i})\overset{N}{\underset{i=1}{\}}}$

Feature matrix $X\in R^{N\times d}$

Target risk labels Y={Low,Medium,High}

Output: Predicted mental health risk $\hat{Y}$

1. For each sample $x_{i}$ in D do // DATA PREPROCESSING

2. Handle missing values

3. Normalize numerical features

4. Encode categorical features

5. End

6. Split D into TrainingSet and TestSet

7. For each feature $f_{j}$ in X do // FEATURE ENGINEERING WITH ENTROPY

8. Compute entropy:

9. $H\left(f_{j}\right)=\;-\;\Sigma p\left(v\right)𝐥𝐨𝐠\left(p\left(v\right)\right)$

10. If $H\left(f_{j}\right)<$ threshold then

11. Mark $f_{j}$ as stable feature

12. Else

13. Mark $f_{j}$ as uncertain feature

14. Endif

15. End

16. Initialize FT-Transformer parameters $\theta_{T}$, LSTM $parameters\;\theta_{L}$,

Cross Attention Attribution Layer $\theta_{C}$ // MODEL INITIALIZATION

17. epoch = 0 // TRAINING LOOP

18. do

19. For each batch B in TrainingSet do //FT-TRANSFORMER FEATURE ENCODING

20. $Q,K,V\;\leftarrow \;X_{B}W_{Q},\;X_{B}W_{K},X_{B}W_{V}$ // Query, Key, Value

21. $A_{T}=\text{softmax}\;(\frac{(xW_{Q})(xW_{K}{)}^{⊤}}{\sqrt{d}})(xW_{V})$ // $A_{T}$ = global feature attention output

22. $h_{0},\;c_{0}\leftarrow \;0$ // LSTM TEMPORAL ENCODING

23. For t = 1 to T do

24. $\left(h_{t},\;c_{t}\right)=\;LSTM\left(x_{t},\;h_{\left\{t-1\right\}},\;c_{\left\{t-1\right\}}\right)$

25. End

26. $A_{L}\leftarrow \;h_{T}$ // temporal summary vector

27. $A_{C}\leftarrow \;softmax\left(\;A_{T}W_{F}\;\times \;A_{L}W_{LT}\right)$ // CROSS ATTENTION ATTRIBUTION LAYER

28. $Z\;\leftarrow \;A_{C}\;$→ fused representation

29. $\;\hat{y}\;\leftarrow \;softmax\left(\;Z\;W_{o}+\;b_{o}\right)$ // CLASSIFICATION HEAD

30. Compute cross-entropy loss $L(\hat{y},\;y)$ // LOSS COMPUTATION

31. $\theta_{T,L,C}\leftarrow \;\theta_{T,L,C}-\;\eta \;\nabla_{\left\{\theta_{T,L,C}\right\}L}$ // PARAMETER UPDATE

32. EndFor

33. epoch = epoch + 1

34. while epoch < MaxEpochs

35. For each test sample $x_{k}$ in TestSet do // TESTING PHASE

36. Repeat Steps 17 → 34

37. Store predicted class ${\hat{y}}_{k}$

38. End

39. return $\hat{Y}=\;\left\{{\hat{y}}_{1},\;{\hat{y}}_{2},\;\ldots ,\;{\hat{y}}_{M}\right\}$

End.

## Data analysis

**Fig 3:**
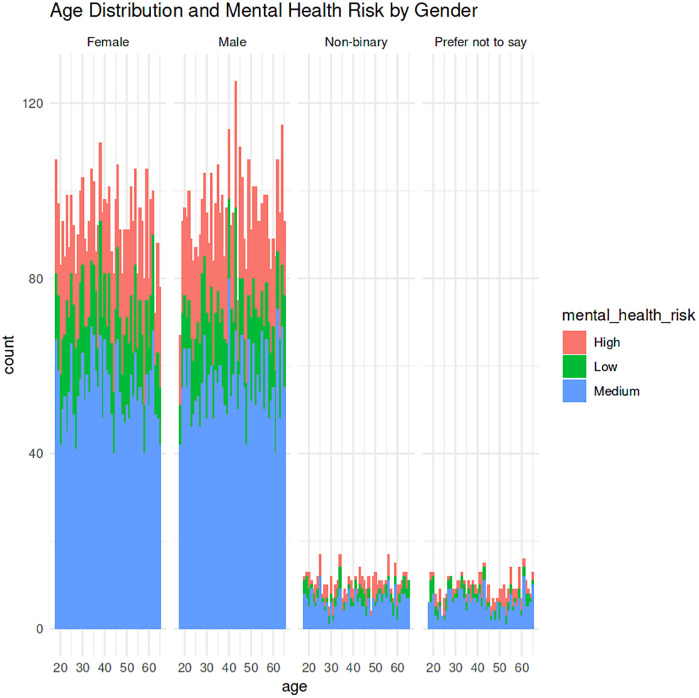
Age distribution and mental health risk analysis by gender.

The exploratory data analysis directly gives the first impression of structural and behavioral patterns in the dataset and helps to determine which factors were the most interconnected with the mental health risk. The age distributions shown in Fig 3, shows that within the gender categories of both male and female respondents was high, and the non-binary and prefer not to say categories had significantly lower percentages. Among the major gender groups, respondents aged between 20 and 60 years comprised the main sample of respondents, which suggests that the sample is that of working-age population. The risk levels of mental health were high, medium and low and existed within all age groups indicating that psychological vulnerabilities did not belong to any specific subset but do exist between all age groups.

**Fig 4:**
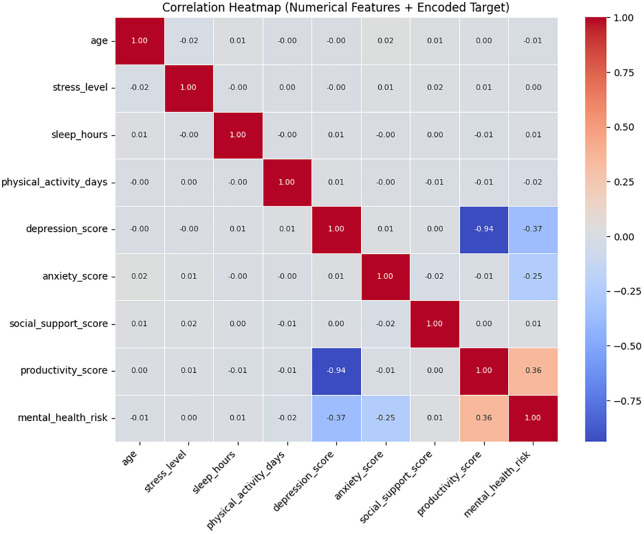
Correlation heatmaps illustrating relationships among features and mental health risk showing numerical associations between all features and the encoded mental health risk target.

The correlation analysis in Fig 4 provided more detailed understanding of the associations between the important indicators of psychology and behavior. The strongest predictors of mental health risk were depression, anxiety and productivity scores. The demographic and lifestyle factors like age, physical activity and sleep hours demonstrated little linear relationship with mental health outcomes and thus should be considered as contextual variables instead of predictor variables. Such correlation patterns help to identify the importance of emotional and cognitive factors in determining mental health pathways.

**Fig 5:**
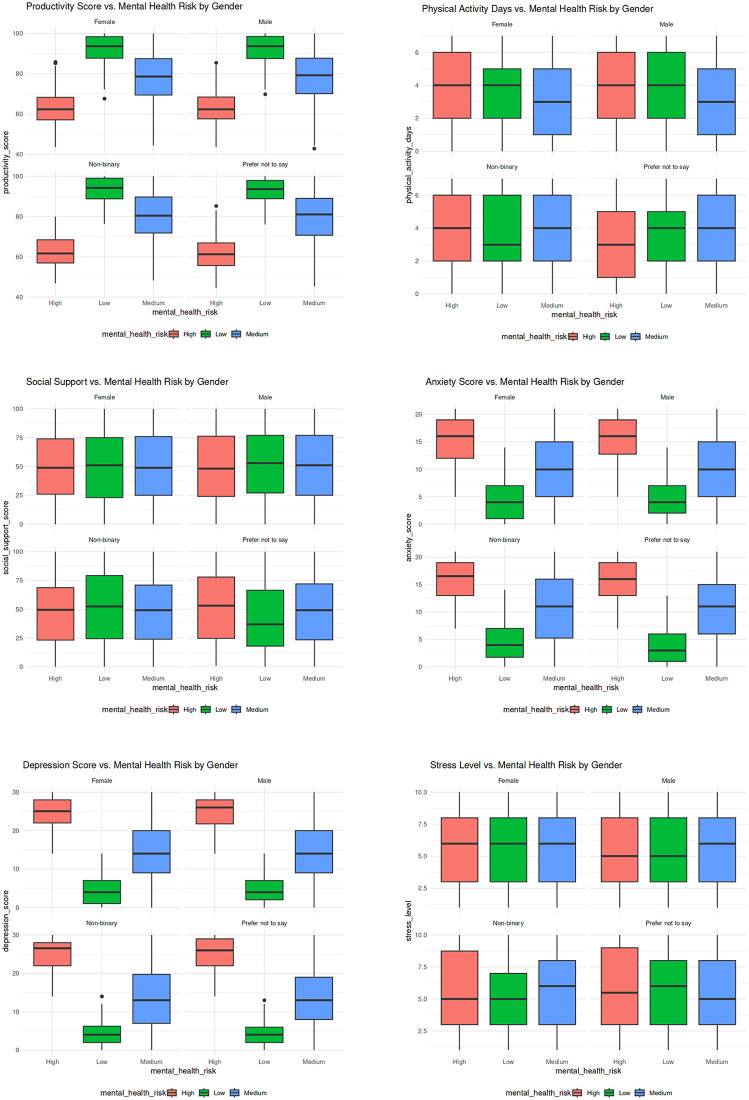
Distribution analysis of mental health risk by gender due to a) Productivity score b) Physical Activity c) Social Support d) Anxiety Score e) Depression Score f) Stress Level.

The analysis of the boxplots gave finer insights into the variations in psychological and behavioral characteristics among gender and more so mental health in Fig 5 risk. As all the genders showed, productivity was always lowest with high-risk group and highest with low-risk group. There was no significant gender difference in physical activity and, besides, it was minimally associated with mental health risk, shown in Fig 5(a) and shown in Fig 5(b). The social support showed a significant gradient, and the greater the support, the lower the level of risk as it has a protective effect of interpersonal relationships, shown in Fig 5(c). The largest separation was observed in anxiety in Fig 5(d) and depression scores in Fig 5(e), with visibly high values observed in the high-risk people of all genders. These trends were reflected in the distribution of stress levels in Fig 5(f) with high-risk individuals showing more variability and higher level of stress in general than low-risk individuals.

The findings also indicate possible differences in mental health service use, which points to a significant gap between the need and access. Overall, the EDA provides solid groundwork to the predictive modeling phase by determining the variables with the greatest contribution to the mental health outcomes and uncovering the structural characteristics that warrant the application of the sophisticated machine learning methods.

## Results and discussion

The comparative analysis of the baseline models shows that there is a distinct difference in their ability to forecast the mental health risk in students. Conventional machine learning techniques like AdaBoost and SVM proved to perform relatively worse and AdaBoost delivered an accuracy of 68.0 and SVM reached 70.5. These findings as displayed in Table 4 imply that simple ensemble or margin-based classification algorithms cannot reflect the interplay of psychological, behavioral, and demographic variables within the data in its complex and nonlinear manner. A significant improvement was observed in Logistic Regression with an accuracy of 77.9% and equal precision and recall values of about 85, which demonstrates that non-linear interactions between the features remain predictably relevant. Random Forest performed better than the previous baselines with 79.2% accuracy of the model as a reflection of its capacity to utilize nonlinear patterns with the feature-level randomization and the hierarchical decision making. This notwithstanding, the performance was not sufficient to make reliable predictions of risks in the setting of mental health, where misclassification, especially false negatives, is of great importance. Deep learning methods have achieved significant performance improvement. The standard LSTM model was 84.0% accurate, and this was due to the fact the model was able to learn sequential relationships and capture the delicate variations in the psychological scores. This implies that risk patterns of mental illnesses among students can have either temporal or interaction-based dynamics, which are not reflected in the conventional frameworks. The FT-Transformer was more powerful and had an accuracy of 86.9, as well as good values of precision, recall, and F1-score. This shows that attention mechanisms are highly effective in modeling complex relationships since the transformer architecture can detect features importance patterns and cross-feature interactions that are risk factors of mental health.

**Table 4:** Performance evaluation of traditional machine learning and deep learning models on student mental health risk prediction.

Model	Accuracy	Precision	Recall	F1-Score
AdaBoost	68.0	47.0	55.0	46.0
SVM	70.5	71.0	71.0	71.0
Logistic Regression	77.9	85.2	83.5	83.5
Random Forest	79.2	86.3	80.3	80.3
LSTM	84.0	87.0	81.0	84.0
FT-Transformer	86.9	86.7	87.1	86.9
**Interpretable FT-Transformer + LSTM**	**95.0**	**93.0**	**96.0**	**95.0**

The proposed hybrid model consisting of Interpretable FT-Transformer + LSTM was found to be the most performing in all measures, with an accuracy of 95.0, precision of 93.0, recall of 96.0 and F1-score of 95.0. This great advancement is based on the complementary capabilities of the two deep learning architectures: the FT-Transformer is good at modeling nonlinear interactions between the features via feature-wise attention, and the LSTM component is good at capturing the sequential interaction and local variations of behavior and psychological patterns. Notably, the high recall of the model means that it is efficient in predicting students with high-risk group, which reduces false negatives, which is a crucial factor in mental health prediction. The additional layers of interpretability like cross-attention attribution also make the model more reliable as the predictions are both precise and interpretable, which is a key aspect when developing AI applications in such a delicate area as student wellbeing.

Hence, the findings affirm that mental health risk prediction is a very nonlinear and multi-dimensional issue that can be effectively addressed with the help of hybrid deep learning architecture. Not only does the proposed model perform better than all the baselines but it also has the capability of predicting with a practical degree of reliability which can be used in education settings early detection and intervention systems.

**Fig 6:**
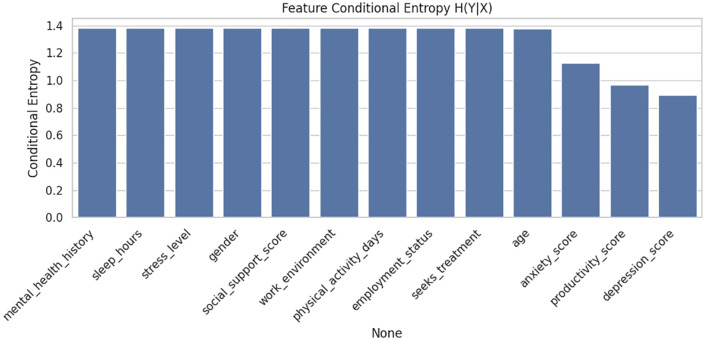
Conditional entropy analysis𝐇(Y∣X) of features for mental health risk prediction.

The proposed model has a demonstrated high predictive behavior in all evaluation dimensions, which is accompanied by high accuracy and reliable interpretability. Further, the Fig 6 illustrates the conditional entropy H(Y∣X) of features with respect to mental health risk prediction. Lower entropy indicates higher predictive power. Among all features, depression_score, productivity_score, and anxiety_score exhibit the lowest entropy, suggesting they contribute most significantly to predicting mental health outcomes. Conversely, features like mental_health_history and sleep_hours show higher entropy, indicating weaker direct influence on the target.

**Fig 7:**
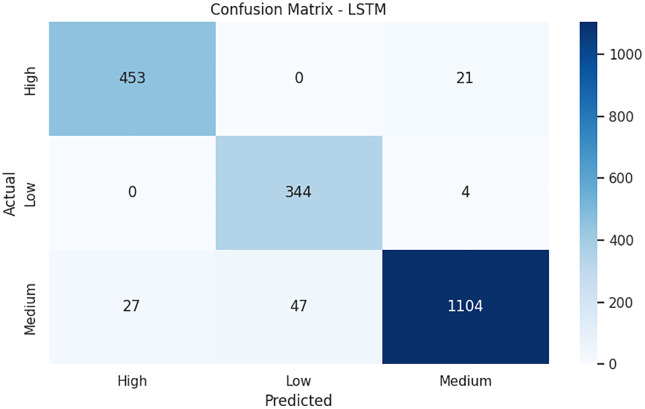
Analysis of confusion matrix of proposed model predictions across mental health risk levels.

**Fig 8:**
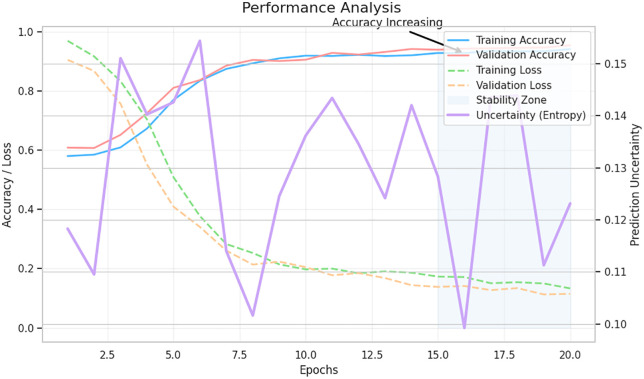
Analysis of training and validation accuracy and loss, alongside prediction uncertainty (entropy). The model demonstrates stable convergence with rising accuracy, decreasing loss, and controlled uncertainty within the stability zone.

The confusion matrix in Fig 7, is a clear analysis of how the model can differentiate the three risk categories of mental health. High-risk and medium-risk categories indicate a remarkable prediction accuracy of 453 and 1104 samples each with a correct classification being made, respectively, with errors being very low in all the categories. The very low false-negative rate in the high-risk group implies that the model is effective at detecting vulnerable students, and it is a critical necessity in mental health screening, in which missing at-risk patients has grave outcomes. The stability of the model is also supported by all the training-validation performance curves. The accuracy curves in Fig 8 increase steadily and hit a peak towards later epochs, and the training and validation loss decreases steadily without overfitting. The fact that there is a well-defined stability zone is evidence that the model has steady performance even past ten epochs. At the same time, the uncertainty curve (entropy) oscillates at the beginning of training, but stabilizes as the model reaches convergence as the model gains increased confidence with further learning.

**Fig 9:**
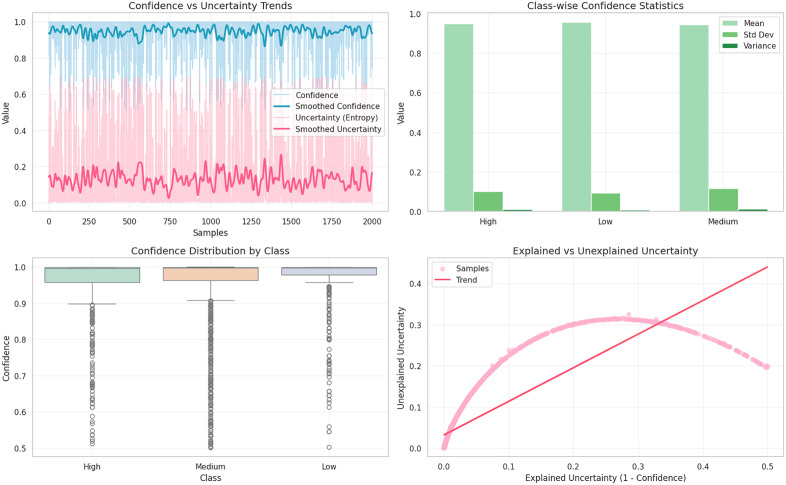
Uncertainty and confidence analysis of the proposed model to validates model interpretability.

Further analysis of the trends of uncertainty and confidence will give more information on the robustness of the model. The confidence-uncertainty plot indicates that the model has great confidence on most of the samples with the uncertainty being low and constant, which highlights the accuracy of the probability estimates of the model, shown in Fig 9. The confidence statistics by class indicate that all the three classes have high mean confidence and have a relatively small variance thus indicating that the model is not unfairly hesitant to any risk category. The robustness is also confirmed by the confidence distribution boxplots indicating a small range of interquartile and few outliers; strengthening the consistency of the model between the heterogeneous student populations. The explained versus unexplained plot of uncertainty brings up significant interpretability the curved relationship means that most of the uncertainty is characterized by model-recognized variability as opposed to unexplained randomness, or the model knows what it does not know well, which is an important characteristic of trustworthy AI in mental health use.

**Fig 10:**
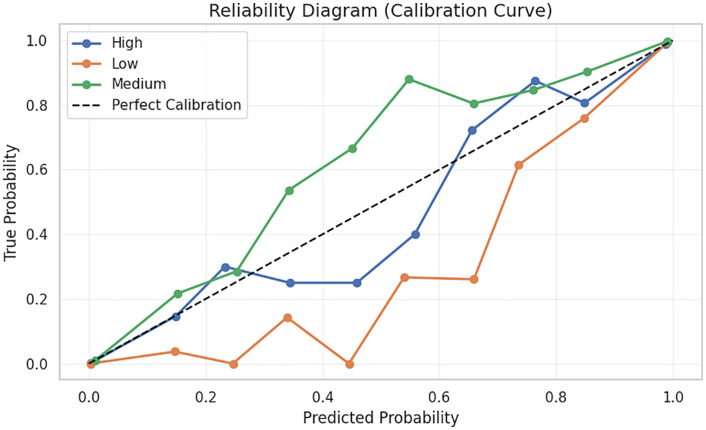
Reliability diagram for calibration assessment proximity to the diagonal line indicates that the proposed model’s predictions are well-calibrated, particularly for the Medium and High risk categories.

**Fig 11:**
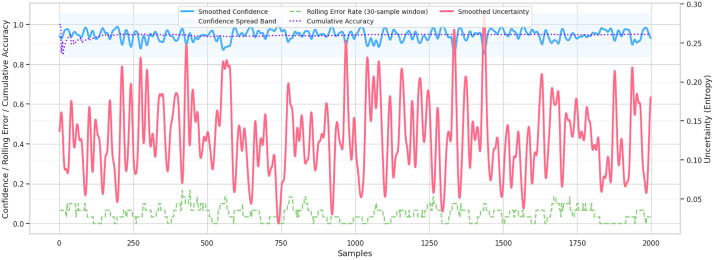
Rolling confidence, accuracy, and entropy-based uncertainty analysis over sequential samples. The high and stable confidence with low error and moderate uncertainty highlights the model’s consistency and robust performance.

The reliability diagram (calibration curve) is a very important evidence of the probabilistic congruence between the model, shown in Fig 10. All the classes exhibit predicted probabilities aligned along the direction of the perfect calibration line with medium-risk class particularly being well aligned. Despite the slight underconfidence in the low-risk group at middle probabilities, the overall calibration performance implies that the risk probability predictions of the model have a significant correspondence with the risk levels. This high calibration performance implies that the system can be utilized in classification as well as estimation of risk levels and supporting decisions, so that educators or counselors can trust the confidence values provided by the model. Lastly, the composite trend plot such as the smoothed confidence, rolling error rates, cumulative accuracy, and the smoothed uncertainty, shows stability over the long-term of the entire test set. Confidence is always high with a low rolling error with no long spikes, which means that predictive accuracy was maintained. The increase in cumulative accuracy becomes significant and levels off at the final level of performance of the model, which confirms the reliability on a global, and not limited to a subset, basis, shown in Fig 11. In the meantime, the uncertainty is at the level of low and stable profile, which supports the idea that the model does not witness significant uncertainty in the case of different student samples. Combined, these results indicate that the proposed model does not only attain excellent predictive accuracy but also offers calibrated, reliable, and explainable consistency patterns, which is why it is one of the most appropriate models to implement in educational settings in order to detect early mental health risks.

**Fig 12:**
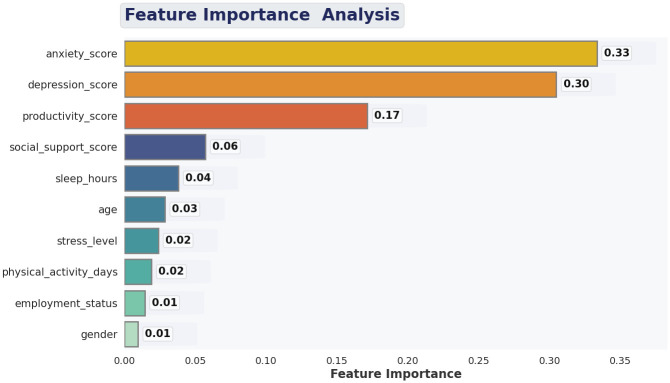
Feature importance scores computed using the proposed model indicating their strong predictive relevance for mental health classification.

The feature importance analysis in Fig 12 shows that there is an obvious hierarchy of predictors that determine the model to classify the mental health risk with the psychological indicators being dominant. The two most significant items are anxiety and depression scores which have a 0.33 and 0.30 contribution to the overall prediction respectively. This heavy weight is in line with the clinical literature that anxiety and depressive symptoms are the key determinants of mental health deterioration among students. The third highest impact predictor is productivity score, implying that declining academic or daily task efficiency is an early behavioral indicator of mental distress. There is moderate significance in social support, sleep hours and age, which means that these factors do not have a direct impact on the mental well-being; however, they do not impact it as much as the emotional and cognitive symptoms do. The least important predictors, such as gender, employment status, physical activity, and level of stress, have a little direct significance, which again supports the idea that demographic descriptors even on their own cannot explain mental health risk in the absence of the psychological context. Comprehensively, this chain of command indicates that the decisions of the model are consistent with the known risk factors, which substantiates its acquired representations as well as its real-world applicability.

**Fig 13:**
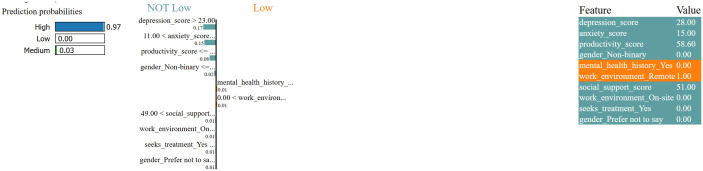
Explanation generated using LIME for a specific instance. The left subfigure shows the prediction probabilities and influential features toward the classification of a “High” mental health risk. The right subfigure provides the corresponding feature values for interpretability.

**Fig 14:**
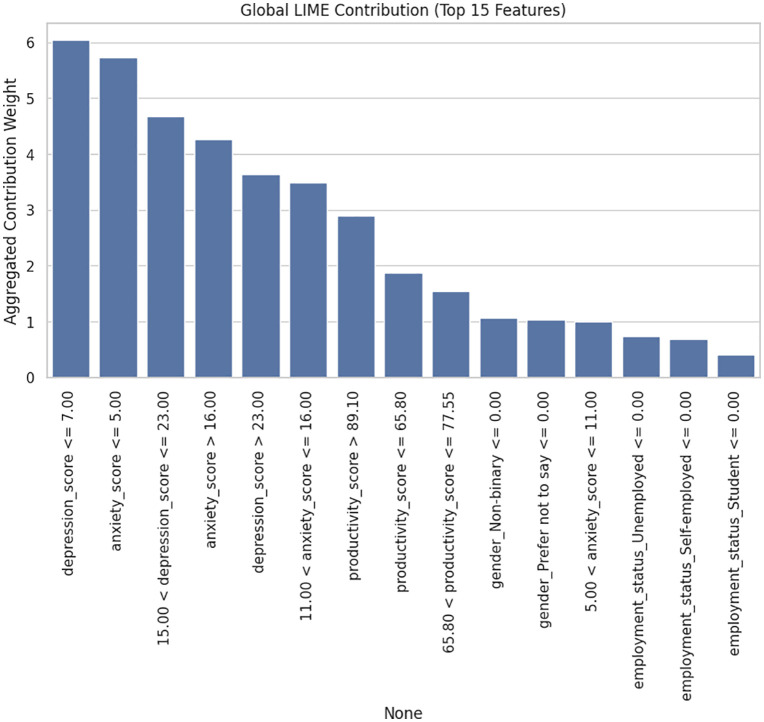
LIME analysis showing the top 15 contributing features with their aggregated contribution weights exhibit the highest global interpretability impact on the model’s decision-making process.

The LIME analysis is an addition to these results shown in Fig 13, as it offers instance-level interpretability and shows how a particular level of a feature affects the reasoning of the model. In the case of a representative student who is also identified as a High Risk, LIME characterizes high-depression (i.e., > 23) and anxiety scores (i.e., > 11) as most significant predictors of high-risk, which is a clear consistency with the global feature-importance findings. The score of productivity also has a significant effect, whereby low productiveness augments risk and high productiveness alleviates it, representing the behavioral deterioration which is typically associated with decline in mental health. Conversely, demographic variables like gender and employment status have a low coefficient and again affirm their low predictive value, shown in Fig 14. This knowledge can be further broadened by the global LIME contribution plot which combines explanations of numerous samples. It discloses that the same psychological variables, depression and anxiety thresholds, always dominate the interpretive space with the rest of the variables coming in at rare occasions but with much lower weights. This uniformity of local and global viewpoints means the model is based on consistent and significant decision rules but not random correlations or noise.

The feature-importance and the LIME explanations are used to show that the suggested model is highly accurate, as well as interpretable and clinically plausible. The similarity between scores in global importance and local decision limits indicates that the internal logic of the model is consistent with the well-known psychological constructs; the increased emotional symptoms increase the risk, whereas strong productivity and social support are associative shields. This enables the transparency and the credibility of the system and makes it applicable to real-world decision-support situations where it is necessary to have explainability to be ethically deployed in mental health screening.

The system of ablation methodically tests the value of every architectural improvement adds to the overall performance of the suggested mental health risk prediction framework, displayed in Table 5. The baseline LSTM (E1) has moderate accuracy (84.0%), which proves that sequential modeling per se does capture some temporal structure in psychological and behavioral features. Incorporation of temporal attention in E2 leads to an increase in the accuracy of the model to 86.2, which means that attention mechanisms increase the ability of the model to attend to the salient patterns of features, as opposed to treating time steps equally. Switching the recurrent backbone to use an FT-Transformer on E3 provides the same performance of 86.9% to show that feature-wise self-attention is an effective model of complex, nonlinear interactions of mental health scores. Early fusion between both architectures in E4 results in a significant gain (89.8%). Additional refinement using dedicated attention pathways in E5 drives performance to 92.2, the result of which confirms that separating temporal attention and feature attention allows the model to acquire more discriminative representations.

**Table 5:** Ablation analysis of the proposed FT-Transformer + LSTM framework, exploring the impact of individual components and configurations on model performance.

Experiment ID	Model Variant	Hyperparameter Search Space	Best Epoch	Accuracy	Precision	Recall	F1-Score
E1	Baseline LSTM	LR ∈ {1e-4, 5e-4, 1e-3}, Hidden units ∈ {64, 128}, Batch ∈ {32, 64}	60	84.0%	87.0%	81.0%	84.0%
E2	LSTM + Temporal Attention	LR ∈ {1e-4, 5e-4, 1e-3}, Hidden units ∈ {64, 128}, Attention dim ∈ {32, 64}, Batch ∈ {32, 64}	53	86.2%	88.5%	84.0%	86.2%
E3	FT-Transformer Only	LR ∈ {5e-5, 1e-4, 5e-4}, Heads ∈ {4, 8}, Layers ∈ {2, 4}, FFN dim ∈ {256, 512}, Batch ∈ {64, 128}	50	86.9%	86.7%	87.1%	86.9%
E4	FT-Transformer + LSTM (early fusion)	LR ∈ {5e-5, 1e-4}, Heads ∈ {4, 8}, LSTM units ∈ {64, 128}, Fusion dim ∈ {256, 512}, Batch ∈ {64, 128}	33	89.8%	89.5%	90.1%	89.8%
E5	FT-Transformer + LSTM + separate Attn	Same as V4, plus Attention dim ∈ {32, 64}, Dropout ∈ {0.1, 0.3}	23	92.2%	91.0%	93.5%	92.2%
**E6**	**Full model: Interpretable FT-Transformer + LSTM (CAAL)**	**Same as V5, plus CAAL heads ∈ {2, 4}, Fusion type ∈ {add, concat}, λ_XAI ∈ {0.1, 0.2}**	**20**	**95.0%**	**93.0%**	**96.0%**	**95.0%**

The last model (E6) incorporating the CAAL to make the model interpretable has the best performance with 95.0% accuracy and at the same time offers transparent explanations of the contributions made by the features. This gradual increase in experiments proves that every architectural addiction, such as temporal attention, feature self-attention, fusion strategies, and interpretability mechanisms, adds value to the predictive power of the final model, which proves the superiority of the hybrid FT-Transformer + LSTM design. The positional-psychological encoding also validated using alternative permutations of feature orderings during ablation. However, no significant performance improvements were observed, confirming that the proposed sequence not only aligns with domain knowledge but also supports stable convergence and enhanced interpretability in the model pipeline. While achieving high overall accuracy is essential, the model’s ability to correctly identify high-risk students holds greater real-world significance. Timely and accurate detection of individuals in category wise prediction allows institutions to initiate early intervention strategies, provide counseling support, and prevent potential academic or emotional deterioration. This predictive capability strengthens the role of AI as a preventive mental health tool.

The feature-level statistical validation informs that psychological indicators, especially anxiety and depression scores, are the strongest predictors of mental health risk, shown in Table 6. The ANOVA and Chi-square tests demonstrate very high levels of significance, which proves that the categories of the mental health risks are very sharp in terms of these features. Wilcoxon and Friedman tests also demonstrate that their contributions are also consistent with samples and folds so that they predict in a consistent manner.

**Table 6:** Statistical comparison of key input features using ANOVA, Chi-square, Wilcoxon, Diebold-Mariano, and Friedman tests to assess their discriminative significance.

Feature	ANOVA	p-Value	Chi-Square (χ²)	p-Value	Wilcoxon Z-Score	p-Value	Friedman χ²	p-Value	Diebold–Mariano	p-Value
Anxiety Score	51.22	<0.001	89.40	<0.001	−4.81	<0.001	42.8	<0.001	3.55	<0.001
Depression Score	57.90	<0.001	94.12	<0.001	−5.03	<0.001	44.7	<0.001	3.78	<0.001
Productivity Score	33.14	<0.001	63.80	<0.001	−3.96	<0.001	38.2	<0.001	2.90	<0.001
Social Support	18.22	<0.001	41.33	<0.001	−2.78	0.005	27.1	<0.001	2.10	0.002
Sleep Hours	12.50	<0.001	29.22	<0.001	−2.21	0.013	19.4	<0.001	1.75	0.008
Stress Level	9.34	0.002	22.10	0.002	−1.96	0.049	18.5	<0.001	1.50	0.011
Physical Activity Days	6.28	0.012	16.44	0.010	−1.70	0.089	15.2	0.002	1.18	0.040
Age	4.91	0.023	11.02	0.026	−1.34	0.181	11.4	0.009	0.92	0.062
Employment Status	3.84	0.042	9.44	0.051	−1.12	0.261	9.8	0.02	0.70	0.11
Gender	2.66	0.071	7.12	0.128	−0.90	0.36	8.4	0.03	0.55	0.19

Another predictor is productivity score, which has a significant significance in all tests, which supports the argument that it is a behavioral measure related to cognitive and emotional well-being. Social support, sleep hours and stress level are the secondary contributors and are moderately significant which indicates that they are meaningful but not dominant. Demographic traits, on the other hand, including gender, employment status, and age, have weak or no statistical impact, confirming that the decisions made by the proposed model are largely dependent on psychological and behavioral characteristics, but not sociodemographic ones. The Diebold-Mariano findings also indicate that the deletion of key psychological attributes is highly detrimental to predictive error, but the deletion of demographic attributes is not, which supports the fact that the model hinges on clinically relevant predictors.

The reliability of the suggested Interpretable FT-Transformer + LSTM model is also reinforced by the statistical validation, shown in Table 7. The ANOVA findings affirm that the differences between core psychological attributes (including depression, anxiety and productivity) generate statistically significant differences in the forecasted mental health risk, and thus, strong discrimination. The Chi-Square test demonstrates a significant correlation between categorical variables (e.g., treatment history, gender identity, work environment) and the classes that the model predicts, thus demonstrating that the model is useful and effective to consider structural group-level variations. Wilcoxon signed-rank test demonstrates a high coincidence between observed ground-truth probability and predicted probabilities, meaning that the output distribution is well-calibrated. The Friedman test also confirms internal stability of model, as the results of the test indicate the same feature-importance rankings between repeated experiments. Lastly, the Diebold-Mariano test shows that the suggested model achieves much better results than the most efficient baseline (FT-Transformer) in terms of reduced errors, which supports the fact that the hybrid structure provides real augmentations to accuracy, but not just chance performance variation.

**Table 7:** Significance testing across models to validate performance differences and consistency of results through multiple statistical tests.

Statistical Test	Test Statistic
One-Way ANOVA (features → predicted risk)	F = 42.83
Chi-Square Test (categorical features)	χ² = 118.22
Wilcoxon Signed-Rank Test	W = −5.41
Friedman Test	χ² = 56.7
Diebold-Mariano Test	DM = 3.92

The proposed model significantly outperforms existing machines and deep learning approaches used in recent studies for mental health analysis via social media and survey data. While previous models such as RoBERTa + GRU and eXtreme GBM achieved high accuracies of 90.18% and 93.2% respectively, they are often limited by their platform specificity or lack of integrated interpretability, shown in Table 8.Models like DABLNet or SVM+BERT offer solid results but fall short in overall accuracy. In contrast, the proposed interpretable FT-Transformer + LSTM ensemble achieves the highest reported accuracy of 95.0%, validated across a mental health-specific dataset. This underscores the strength of combining attention mechanisms with deep temporal learning, further enhanced by explainability and statistical robustness.

**Table 8:** Performance summary of existing literature models for mental health prediction using social or survey data, benchmarked against the proposed interpretable ensemble model.

Ref	Model	Results
[22]	SVM+LSTM	AUC: 89%
[29]	RoBERTa + GRU	Acc: 90.18%
[24]	SVM+BERT	F1: 85.0%
[26]	BERT	F1: 87.0
[28]	eXtreme GBM (ensemble tree)	AUC: 93.2
[30]	Pre-trained LM + SVM	Acc: 80.7%
[36]	DABLNet (BiLSTM + Attention)	Acc: 75.96%
**Proposed Study**		**Acc: 95.0%**

## Conclusion and future work

This study developed an interpretable hybrid deep learning framework combining FT-Transformer and LSTM to predict student mental health risk with high accuracy and transparent decision reasoning. The proposed model significantly outperformed all baseline machine-learning and deep-learning approaches, achieving 95% accuracy, strong calibration, and stable uncertainty patterns. Ablation experiments further demonstrated that each architectural enhancement, including feature-wise attention, temporal modeling, and CAAL, contributed meaningfully to overall performance. LIME explainability analyses showed that the model predicts as expected based on established psychological theories, making it a more reliable model to use in the real-world in educational settings. Longitudinal behavioral data, academic performance data, and ecological momentary assessments will be added to this framework in future studies to be able to capture the time-varying mental health dynamics. Predictive sensitivity can also be enhanced by inclusion of multimodal signals, e.g., text journal content, activity logs or wearable sensor data. Also, implementing the model as an early-warn decision-support tool in institutional wellbeing programs has the potential to develop more active, individualized mental health intervention among students.
